# Performance and Function Meet Structure: A White Matter Connection Tuned for Vocal Production

**DOI:** 10.1089/brain.2018.0627

**Published:** 2018-12-14

**Authors:** John J. Sidtis, Muhammad Asim Mubeen, Ali Asaei, Babak Ardekani, Diana Van Lancker Sidtis

**Affiliations:** ^1^ Brain and Behavior Laboratory, Geriatrics Department, The Nathan Kline Institute for Psychiatric Research, Orangeburg, New York.; ^2^ Department of Psychiatry, New York University Langone School of Medicine, New York, New York.; ^3^ Center for Biomedical Imaging and Neuromodulation, The Nathan Kline Institute for Psychiatric Research, Orangeburg, New York.; ^4^ Department of Communicative Sciences and Disorders, New York University Steinhardt School, New York, New York.

**Keywords:** basal ganglia, caudate, diffusion tensor imaging, laterality, tractography, vocalization

## Abstract

Contemporary imaging techniques have increased the potential for establishing how brain regions interact during spoken language. Some imaging methods report bilateral changes in brain activity during speech, whereas another approach finds that the relationship between individual variability in speech measures and individual variability in brain activity more closely resembles clinical observations. This approach has repeatedly demonstrated that speaking rate for phonological and lexical items can be predicted by an inverse relationship between cerebral blood flow in the left inferior frontal region and the right caudate nucleus. To determine whether morphology contributes to this relationship, we examined ipsilateral and contralateral white matter connections between these structures using diffusion tensor imaging, and we further assessed possible relationships between morphology and selected acoustic measures of participants' vocal productions. The ipsilateral connections between the inferior frontal regions and the caudate nuclei had higher average fractional anisotropy and mean diffusivity values than the contralateral connections. Neither contralateral connection between inferior frontal and caudate regions showed a significant advantage on any of the average morphology measures. However, individual differences in white matter morphology were significantly correlated with individual differences in vocal amplitude and frequency stability in the left frontal–right caudate connection. This cortical–striatal connection may be “tuned” for a role in the coordination of cortical and subcortical activity during speech. The structure–function relationship in this cortical-subcortical pathway supports the previous observation of a predictive pattern of cerebral blood flow during speech and may reflect a mechanism that ensures left-hemisphere control of the vocal expression of propositional language.

## Introduction

Spoken language does not simply emanate from a “speech area” in the brain, but it is the result of a coordinated network of multiple cortical and subcortical interactions. Well before functional imaging, 19th-century observations on the effects of focal brain damage reported by Paul Broca postulated a speech production area in the inferior frontal region of the left cerebral hemisphere. The consistent localization of propositional speech and language (i.e., rule-governed expressions for generating an unlimited number of novel utterances using phonological, syntactic, and lexical processes; Chomsky, 1957) to the left cerebral hemisphere of right-handed individuals has been a cornerstone of clinical and behavioral neurology (Davis and Wada, 1978).

The abnormally formed speech (dysarthria) that accompanies disorders such as Parkinson's disease and the cerebellar ataxias added pieces to the puzzle, revealing the importance of the basal ganglia and cerebellum for normal speech. Neurological disorders sketch a network for speech production, whereas functional imaging offers the potential for creating a more detailed blueprint.

As part of the developing blueprint for the speaking brain, we previously identified a simple, reliable pattern of blood flow changes with positron emission tomography (PET) that is predictive of speech rates during functional imaging. In normal speakers (Sidtis et al., 2003; replicated in 2018) and in individuals with hereditary spino-cerebellar ataxia (SCA; Sidtis et al., 2006), blood flow increases in the left inferior frontal region of the cerebral cortex and decreases in the head of the right caudate nucleus of the basal ganglia in a linear relationship with speech rate. In SCA, this relationship was observed for at least a 2-year period of disease progression (Sidtis et al., 2010). In Parkinson's disease, this relationship takes a different form and is modified by therapeutic deep brain stimulation of the subthalamic nucleus (Sidtis et al., 2011).

The changes in the left inferior frontal region and the right caudate nucleus during speech observed with functional imaging correspond to clinical observations in people with left or right hemisphere damage (Davis and Wada, 1978; Caplan et al., 1990). Also consistent with this cortical–striatal relationship, many examples of motor planning and execution have been described for the basal ganglia (Divac and Öberg, 1979; Marsden, 1982; Takakusaki et al., 2004). The caudate nucleus, in particular, has been seen as playing a key role in control of vocalized expression (Bhatia and Marsden, 1994). This study used white matter imaging to explore the possibility that there is a morphological component to the functional cortical–striatal relationship reliably observed in cerebral blood flow during spoken verbal expression.

In part, this study was motivated by evidence of “tuning” for specific processes in the nervous system. For example, electrophysiological responses in the auditory brainstem (Song et al., 2008) and cortex (Bao et al., 2004) have been documented to modify in response to auditory experience. Rather than being hard-wired, these and other reports describe neural mechanisms as experience dependent, displaying “intrinsic plasticity with function” (Tzounopoulos and Kraus, 2009, p. 465). In addition to electrophysiological evidence of plasticity, there is a growing body of work that demonstrates structural gray and white matter plasticity in response to experience (Draganski et al., 2006; Draganski and May, 2008). Expanding this work, the characteristics of white matter pathways in the temporal lobe have been shown to be related to performance on a semantic learning task (Rispollés et al., 2017).

In this study, measures of stability in vocalized expression were used as behavioral/performance measures to explore possible relationships with the white matter characteristics of the ipsilateral and contralateral cortical–striatal pathways. As the acoustics of vocal production show great individual differences, being influenced by many factors including gender, age, and physical characteristics, we used acoustic measures of stability as our behavioral target. Acoustic stability measures have been successfully used in mapping cerebral activity during vowel production (Sidtis, 2015).

Probabilistic tractography measures derived from magnetic resonance diffusion imaging (Behrens et al., 2003, 2007) were used to characterize the ipsilateral and contralateral white matter connections between these brain regions, bilaterally. This technique measures the direction and the magnitude of the diffusion of water molecules in the brain. Both the magnitude and the degree of anisotropy of the diffusion are affected by anatomical constraints, particularly in the white matter. Regions of interest (ROIs) were drawn for the inferior frontal regions and the heads of the caudate nuclei for each subject to account for individual differences in anatomy. Two fundamental questions were examined. First, is the crossed pathway linking the left inferior frontal region and the head of the right caudate nucleus morphologically different from the other inferior frontal-caudate connections? Second, are the morphological characteristics of the two contralateral and ipsilateral pathways related to acoustic stability of the phonated portions of vocalized speech? This second question was addressed by examining the relationships between individual differences in morphological measurements with individual differences in vocal stability.

## Methods

### Participants

The contralateral and ipsilateral connections between the left and right inferior frontal regions and the left and right caudate nuclei were examined in a group of 29 normal, adult, right-handed, native English-speaking individuals (mean age = 60 ± 10.7 years; 22 females, 7 males) by using diffusion tensor imaging data and structural images. These people were recruited from the Nathan Kline Institute (NKI)-Rockland Sample imaging project (Nooner et al., 2012) to participate in a separate study of speech production. We have studied speech and cerebral blood flow in this age group in our studies of normal function, SCA, Parkinson's disease, and deep brain stimulation. Subjects provided informed consent in accordance with the NKI/Rockland Psychiatric Center Institutional Review Board and the Helsinki Declaration of 1975 (and as revised in 1983).

### Brain imaging

All magnetic resonance images (MRI) were acquired by using a Siemens 3T scanner as part of a large-scale study of normal subjects (Nooner et al., 2012). Three-dimensional (3D), high-resolution, whole-brain images were obtained for each participant by using an magnetization prepared rapid gradient-echo (MPRAGE) sequence. The following acquisition parameters were used: repetition time (TR) = 1900 ms, echo time (TE) = 2.52 ms, inversion time = 900 ms, flip angle = 9°, measurements = 1, field of view (FOV) = 250 mm, matrix size = 256 × 256, voxel size = 1.0 × 1.0 × 1.0 mm^3^, and slice thickness = 1 mm. Diffusion weighted images (DWI) consisted of 137 volumes, 128 acquisitions with diffusion-sensitizing gradient directions with diffusion weighting of *b* = 1500 s/mm^2^ and 9 acquisitions without diffusion weighting (i.e., *b* = 0 s/mm^2^). The following acquisition parameters were used in DWI: TR = 2400 ms, TE = 85 ms, flip angle = 90°, measurements = 1, FOV = 212 mm, base resolution = 106, voxel size = 2.0 × 2.0 × 2.0 mm^3^, and slice thickness = 2 mm.

### Image processing

The structural MPRAGE images were used for pre- and post-tractography processing. The preprocessing of structural images began with automatic skull-stripping by using the Brainwash software from the Automatic Registration Toolbox (Ardekani, 2008; Ardekani and Bachman, 2009). Some manual corrections were performed by using ITK-SNAP (Yushkevich et al., 2006). For the probabilistic tractography, ROIs for the seeds (head of left and right caudate) and targets (left and right inferior frontal region) were manually specified by using ITK-SNAP in individual structural images. The selection of brain regions is critical to many approaches to image analysis. Although automatic selection might seem ideal, techniques for alignment and spatial normalization are not perfect and individual differences in anatomy are significant. There are no distortion-free methods that accommodate the variability inherent in these anatomic differences (Tonga and Thompson, 2002). The investigators have significant experience identifying the seed and target regions in multiple publications using PET images. The head of the caudate is easily visualized, and the inferior frontal region is identified by using the Sylvian fissure and the anterior tip of the temporal lobe as landmarks. This minimizes the opportunity for bias as there is no visualized information regarding connectivity at the stage of processing at which the regions are drawn. To utilize these ROIs in the tractography process, structural images were nonlinearly registered to DWI by using FSL software library (Behrens et al., 2003, 2007). An inverse registration was performed to return the tractography results to structural space to perform various post-tractography calculations. DWI were also preprocessed for probabilistic tractography by using FSL (Behrens et al., 2003, 2007). DWI volumes were skull-stripped by using an FSL utility named “BET” (Smith, 2002). Diffusion tensors were estimated by using a utility named “dti_fit.” This utility calculated diffusion tensors by using least-square estimation to the log of diffusion signal.

### Bayesian probabilistic tractography

**FIG. 1.**
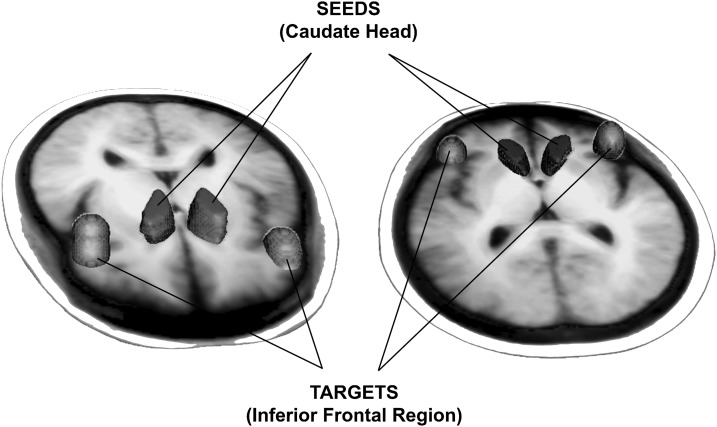
For probabilistic tractography, volumes of interest for the seeds (head of left and right caudate) and targets (left and right inferior frontal region) were manually created for each individual structural image. Composite seeds and targets across all individuals are depicted in two orientations for clarity. Structural images were nonlinearly registered to diffusion weighted images; then, a reverse registration was performed to return the tractography results to structural space to perform post-tractography calculations.

Probabilistic estimates of the white matter tracts were computed by using manually drawn seeds (left and right head of the caudate) and targets (left and right inferior frontal areas) for each individual (Fig. 1). Subject-specific seeds and targets were used to deal with individual differences in anatomy. Three measures were examined: volume of the white matter tracts, fractional anisotropy (FA, a measure of white matter integrity based on the degree to which water diffusion is constrained by the density, size, and myelination of axons), and mean diffusivity (MD, an estimate of tissue density).

After preprocessing the structural and diffusion images, diffusion parameters were estimated by using Bayesian estimation of diffusion parameters obtained by using sampling techniques BEDPOSTX (Behrens et al., 2007), with the X indicating the ability for model crossing fibers. The crossing fiber option was not used. BEDPOSTX creates a platform to perform probabilistic tractography by creating distributions of diffusion parameters at each voxel. Probabilistic tractography was performed by using the seed and target ROIs defined in the structural images and registered to corresponding diffusion images using the FSL utility “probtrackx” (Behrens et al., 2003; 2007). This utility repetitively samples from the distribution of principal diffusion directions for each voxel, computing a streamline through these samples to generate a probabilistic model. By repetitive sampling, a histogram of the connectivity distribution is created and stored as a 3D image.

The tractography analysis was bound by the seed (left or right head of the caudate nucleus) and target (left or right inferior frontal region) ROIs and carried out by using FSL's routine “probtrackx” for all combinations of seeds and targets. This routine was run with the following parameters: number of iterations per voxel was 5000, maximum number of steps for a streamline was 2000 with the step size 0.5 mm, curvature threshold was 0.2, and the modified Euler's streamlining option was used. Two output files from this routine were used. The “fdt_paths.nii” file is a 3D image containing the output connectivity distribution between the seed and target ROIs. The text file “waytotal” contains one number per seed mask corresponding to the total number of tracts generated from each seed ROI that have successfully reached the target ROI. “Probtrackx” was run for one seed and one target at a time, so the “waytotal” file contains one number and “fdt_paths.nii” file is a 3D connectivity distribution image for a specific set of seed and target.

Post-tractography calculations of connectivity and diffusion measures were performed by using the 3D image file for the tracts. As individual structural images were used to define the seed and target ROIs, the sizes of these ROIs varied for different participants and resulting path files could have been dependent on ROI size. To compare the results across participants, we divided the “fdt_paths.nii” data by the corresponding waytotal values and employed a threshold so that the normalized 3D images of the fdt_paths represented the upper 90% of the data. This provided a binary mask based on the tract probability. The volume for the normalized path calculated in this way was determined. The binary mask in structural space was registered to FA and MD 3D images, respectively. The mean FA and mean MD values were then calculated for the normalized tracts. FA values <0.2 were excluded.

### Speech samples

Participants for the speech study were recruited from the group who underwent MRI as part of the study independent of the NKI-Rockland Sample Project. Speech tokens previously used to identify the cortical–striatal speech network (Sidtis et al., 2003, 2006, 2010, 2011, 2018) were recorded at separate sessions on different days after MRI acquisition. Part of the protocol evaluation consisted of repeating single consonant-vowel syllables (/*pa*/, /*ta*/, and /*ka*/) and a syllable sequence (/*pa-ta-ka*/) as quickly as possible on a single breath. Syllable repetitions were digitally recorded for subsequent analyses. Recordings were made by using a Marantz Professional digital recorder (PMD660) and a Shure unidirectional head-worn dynamic microphone (SM10A). Simultaneous backup recordings were made with a separate PMD660 digital recorder and a boom-mounted AKG D5 microphone. All recordings were made in .wav format at a 48k sampling rate. The grooved pegboard (Ruff and Parker, 1993), a non-speech digit dexterity test, was administered to both hands as a non-vocal comparison condition as was done in a study of basal ganglia morphology in childhood stuttering (Foundas et al., 2013).

Acoustic analyses quantified vocal amplitude stability (shimmer) and pitch stability (jitter) in the vocalic portion of each syllable. These served as the performance measures of vocal production. A non-speech comparison task requiring fine motor control assessed dexterity for each hand (Ruff and Parker, 1993). The frequency and amplitude stability of the vowel portions of the repeated syllables were analyzed by using PRAAT (Boersma and Weenick, 2009). Frequency stability was quantified by using several measures of cycle-to-cycle fluctuations in the frequency period of the waveform: Jitter (local—2 periods), the average absolute difference between consecutive periods divided by the average period; Jitter (RAP—3 periods), the average absolute difference of one period from the average of its two neighbors, divided by the average period. Amplitude stability was quantified by using several measures of cycle-to-cycle fluctuations in the amplitude of the waveform: Shimmer (local—2 periods), the average absolute difference between consecutive periods divided by the average period; Shimmer (apq 3 periods), the three-point amplitude quotient, the average absolute difference between the amplitude of a period and the average amplitude of its neighbors, divided by the average amplitude; Shimmer (apq 11 periods), the 11-point amplitude quotient, the average absolute difference between the amplitude of a period and the average amplitude of its ten closest neighbors, divided by the average amplitude (Teixeira et al., 2013).

### Statistical analysis

Measures of white matter characteristics were analyzed by using mixed-design repeated-measures analyses of variance with a full-factorial model, and paired *t*-tests as appropriate. Spearman's correlations were used to examine the relationships between white matter characteristics and speech performance measures as the MR and speech data have different scale properties (SPSS for PC version 7.5). Probability values (*p*) <0.025 (two-tailed) in the correlation analyses were considered significant.

## Results

### Morphological characteristics of left/right, ipsilateral/contralateral connections

**FIG. 2.**
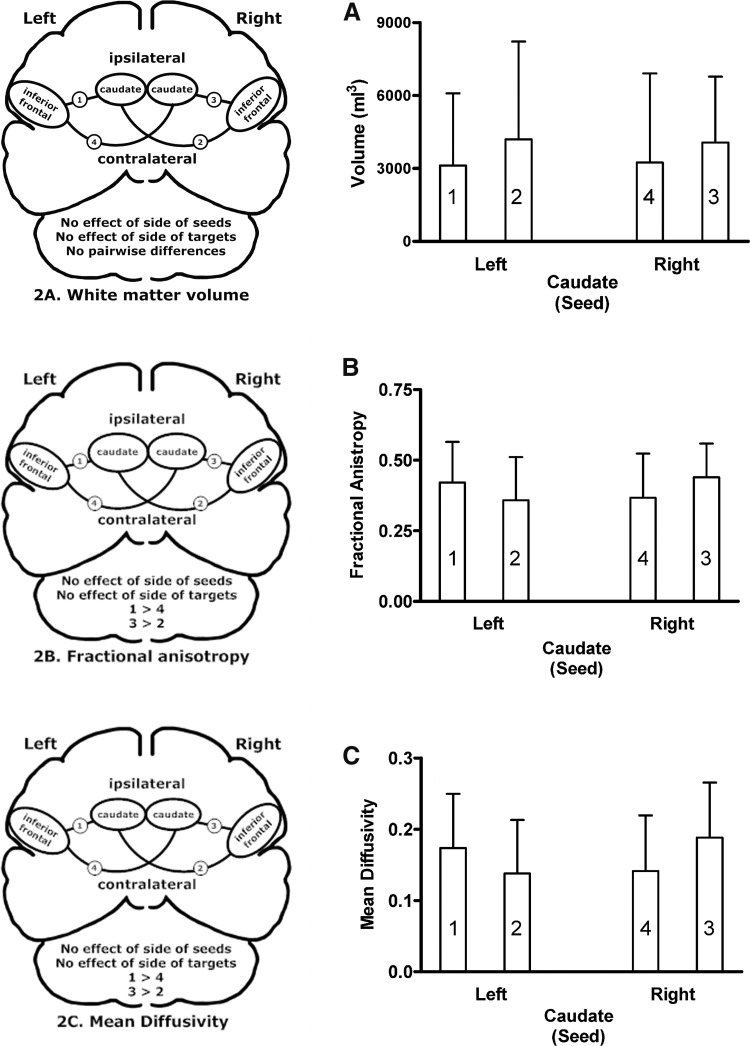
Brain schematics and group means and standard deviations for ipsilateral (connections 1 and 3) and contralateral (connections 2 and 4) pathways between the inferior frontal regions and the left and right caudate. **(A)** Represents white matter volumes; **(B)** Represents fractional anisotropy measures; and **(C)** Depicts the mean diffusivity measures.

Analysis of normalized white matter volumes revealed large between-subject variability and no significant main effects of laterality for seeds or targets, no interaction between seeds and targets, and no pairwise differences involving laterality or ipsilateral–contralateral comparisons (Fig. 2A). There were no significant main effects of laterality for seeds or targets on FA (Fig. 2B), but they did interact [*F*(1,28) = 12.17; *p* = 0.002]. The ipsilateral left inferior frontal–left caudate connection had higher FA than the contralateral left inferior frontal–right caudate connection [*t*(28) = 2.57; *p* = 0.016]. Similarly, the ipsilateral right inferior frontal–right caudate connection had higher FA than the contralateral right inferior frontal–left caudate connection [*t*(28) = −2.54; *p* = 0.017]. The MD demonstrated comparable results (Fig. 2C). There were no significant main effects of laterality for seeds or targets, but they did interact [*F*(1,28) = 14.27; *p* = 0.001]. The ipsilateral left inferior frontal–left caudate connection had higher MD than the contralateral left inferior frontal–right caudate connection [*t*(28) = 2.83; *p* = 0.008]. The ipsilateral right inferior frontal–right caudate connection had higher MD than the contralateral right inferior frontal–left caudate connection [*t*(28) = −2.89; *p* = 0.007]. In general, the ipsilateral connections had higher FA and MD values than the contralateral connections, but the answer to the first question posed in this study is that none of the group average white matter structural characteristics distinguished the left inferior frontal–right caudate connection from the other pathways.

### Relationships between white matter characteristics and vocal acoustics

Potential relationships between the acoustic characteristics of the speech samples from each participant, as measured at the vocalic portions of syllables, and their normalized white matter volumes, their FA values, and their MD measures for each of the four cortical–striatal tracts were examined by using Spearman's correlations. The results of this analysis addressing individual differences in speech and brain morphology are presented in Table 1. Significant relationships between the characteristics of the contralateral left inferior frontal–right caudate connection were found with shimmer (amplitude) and jitter (frequency) perturbations for each of the syllable production conditions. Associations with white matter structure were slightly more prevalent with frequency perturbations (50% of the possible relationships were significant) compared with amplitude perturbations (39.5% of the possible relationships were significant). It should also be noted that the structural features were associated with local perturbations, that is, those measured over two or three periods for both amplitude and frequency. When local perturbations were considered, 54% of the possible correlations with shimmer and 50% of the possible correlations with jitter were significant. Further, the correlations were all positive, indicating that greater instability in the acoustic measures was associated with greater white matter structure. None of the other connections was associated with acoustic measures, and none of the characteristics of the examined pathways was associated with manual dexterity performance.

**Table 1.** The Relationships Between Inter-Subject Variability in White Matter Structure and Acoustic Stability

*Structural measure*	*Acoustic measure*	*/pa/*	*/ta/*	*/ka/*	*/pa-ta-ka/*
	Shimmer (2 periods)	—	—	**0.514** (0.009)	0.461 (0.02)
Volume	Shimmer (3 periods)	—	**0.539** (0.005)	**0.58** (0.002)	0.449 (0.024)
	Shimmer (11 periods)	—	—	—	—
	Jitter (2 periods)	**0.544** (0.005)	**0.678** (< 0.001)	**0.564** (0.003)	**0.554** (0.004)
	Jitter (3 periods)	0.461 (0.02)	**0.673** (< 0.001)	**0.634** (0.001)	**0.567** (0.003)
Mean diffusivity	Shimmer (2 periods)	**0.522** (0.007)	—	—	—
	Shimmer (3 periods)	0.499 (0.011)	—	—	—
	Shimmer (11 periods)	0.462 (0.02)	—	—	—
	Jitter (2 periods)	—	—	—	0.475 (0.016)
	Jitter (3 periods)	—	—	—	—
Fractional anisotropy	Shimmer (2 periods)	**0.56** (0.004)	—	—	—
	Shimmer (3 periods)	**0.538** (0.006)	0.457 (0.022)	0.457 (0.022)	—
	Shimmer (11 periods)	0.45 (0.024)	—	—	—
	Jitter (2 periods)	0.477 (0.016)	0.449 (0.024)	—	0.457 (0.022)
	Jitter (3 periods)	—	—	—	—

This table presents the significant Spearman's correlations between acoustic stability measures and the characteristics of the connections between the caudate (seed) and the left inferior frontal region (target). Correlations with probability values less than 0.01 are presented in bold. Shimmer is a measure of cycle-to-cycle amplitude stability using 3, 11, or all points as reference. Jitter is a measure of cycle-to-cycle frequency stability. There were no correlations involving the remaining two pathways.

## Discussion

The group average tractography data alone did not distinguish the left inferior frontal–right caudate connection from the other pathways. However, the analyses examining the relationship between individual differences in acoustic stability and individual differences in white matter morphology did identify a structure–function correlation in this cortical–subcortical pathway. Subjects with higher levels of vocal frequency and amplitude perturbations were more likely to have larger normalized white matter volumes, greater tissue integrity, and, to a lesser extent, greater tissue density in the white matter connecting the left inferior frontal region and the right caudate, areas repeatedly shown to be associated with speech rate using PET measures of blood flow. The positive relationships between acoustic instability and white matter integrity are interesting. Although it might seem that stronger white matter connections might yield more stable vocal production, alternatively, it can also be speculated that the stronger white matter connections could reflect structural adaptations in individuals for whom vocal stability requires greater control. Although its functional significance remains to be clarified, the white matter connection between the left inferior frontal region and the head of the right caudate nucleus appears to be tuned to intrinsic features of a planned vocal utterance. Such possible tuning may play a significant role in cortical–striatal interactions that are necessary for proper execution of a vocal expression.

Tuning is prevalent in sensory systems where neuronal activity is related to specific stimulus features (Patel and Iversen, 2007; Tervaniemi et al., 2006; Allman et al., 1985; Ringach et al., 1997; Wehr and Zador, 2003; Bensmaia et al., 2008), and it has been shown to occur developmentally in the animal model (Johnson et al., 2008; Knudsen, 1998). These observations have also been made in the motor system, where corticomotoneuronal cells are tuned to specific functional uses of a muscle (Cheney and Fetz, 1980; Muir and Lemon, 1983; Hoffman and Strick, 1986; Griffin et al., 2015). In sensory systems, neuronal tuning is likely integral for perception. In the motor system, neuronal tuning is associated with the execution of specific motor acts. The present results suggest tuning in a cortical–striatal white matter connection for vocal production. As the relationships between acoustic measures and white matter structure are likely based on the activity of the left inferior frontal region, a cortical speech area, rather than from sensory or motor feedback areas, the relevant functional connection appears to be based on vocal instabilities that are inherent in the motor program for an individual's speech.

### The right caudate nucleus in speech production

Lesions of either the left or right caudate can result in disordered speech, and this abnormality may be more common after right-sided lesions (Caplan et al., 1990). In aphasic individuals, better speaking ability has been correlated with higher relative glucose metabolism in the left caudate, whereas poorer speaking ability was correlated with higher relative glucose metabolism in the right caudate (Metter et al., 1984). In progressive SCA, more severe dysarthria was associated with higher right caudate blood flow (Sidtis et al., 2006). In a meta-analysis of published basal ganglia lesions, caudate lesions were associated with speech disturbances and abnormalities in behavioral control, either initiation (abulia) or disinhibition, or sometimes both in the same individual (Bhatia and Marsden, 1994). Further, an abnormally small right caudate is associated with stuttering in children (Foundas et al., 2013). Basal ganglia dysfunction has been suggested as a likely cause of stuttering (Alm, 2004), but the nature of the dysfunction has not been identified. These clinical observations demonstrate the importance of the caudate, especially the right caudate, in the coordinated activity of cortical and subcortical regions in the production of speech.

### Facilitation and inhibition in cortical–striatal interactions

The clinical observations regarding the right caudate and speech, together with the present demonstration of a tuned white matter connection with the left hemisphere speech area, suggest a novel concept in cortical–striatal interactions: Ipsilateral and contralateral cortical input to the caudate may have different functional consequences with respect to motor control. For fluent speech, which requires a degree of asymmetrical hemispheric control, left hemisphere input to the right caudate may result in inhibition or disconnection of cortical areas in the right hemisphere from the speech control process. Although possible differences in the roles of ipsilateral and contralateral cortical–striatal connections have not been addressed in models of the basal ganglia, complementary functions for these connections could play a role in maintaining unilateral control of a complex production system such as that involved in speaking.

One characterization of the basal ganglia's role in motor function proposes that these structures provide both a focused selection of a desired motor action and inhibition of competing motor programs (Mink, 1996), a general formulation consistent with basal ganglia involvement in the planning, initiation, and stopping motor activities (Graybiel et al., 1994; Aron and Poldrack, 2006). In this framework, the left striatum could provide focused selection, supporting left hemisphere motor speech programs. Focused selection is also consistent with the left caudate's role in language switching in bilingual individuals (Crinion et al., 2006). In contrast, the right striatum may reflect inhibition of access to vocal production by right hemisphere motor areas during speech. It has been suggested that inhibition in motor control reflects a race between competing basal ganglia pathways (Schmidt et al., 2013). A movement is successfully inhibited when the striatum processes a stop signal before movement initiation reaches a critical point. The need to quickly inhibit right hemisphere motor control of speech structures to maintain fluency may be reflected in the morphology of the connection between the left inferior frontal region and the right caudate: Tuning for the characteristics of the individual's motor speech program could provide a speed advantage for stopping signals, which could favor lateralized cortical control. The clinical studies documenting fluency disorders after basal ganglia dysfunction may represent a failure to inhibit input from the right hemisphere to the speech articulators, reflecting an inadequate cortical–striatal white matter connection, a poorly functioning striatum, or a failure within the right hemisphere basal ganglia complex. The proposed roles of the striatum in motor facilitation and inhibition are not new, but the concept that they may represent intra-hemispheric and inter-hemispheric processes is novel.

Although this formulation of cortical–subcortical control of speech is consistent with the production of phonological and lexical units in propositional speech, normal conversation speech likely represents a more complicated situation. Studies have shown that as much as 25% of normal conversations consist of formulaic expressions, a language mode that differs from propositional speech (Van Lancker and Rallon, 2004; Van Lancker Sidtis, 2015). In a recent replication of the predictive relationship between the production rate for phonological and lexical items and the inverse blood flow relationship between the left inferior frontal region and the head of the right caudate nucleus, we observed a complementary relationship between the proportion of formulaic expressions in conversational speech and an inverse blood flow relationship between the right inferior frontal region and the head of the left caudate nucleus (Sidtis et al., 2018). As with the cortical–subcortical relationship for propositional speech, the complementary cortical–subcortical relationship for formulaic expressions is also consistent with the clinical literature that demonstrates the importance of the right cerebral hemisphere and the basal ganglia (Van Lancker Sidtis and Postman, 2006; Bridges et al., 2013; Van Lancker Sidtis et al., 2016; Van Lancker Sidtis and Sidtis, 2018). Although these observations lead to a more complicated process of coordinating multiple cortical and subcortical brain regions during normal conversational speech, they emphasize the dynamic nature of the functional anatomy of expressive language, which incorporates several modes of expression.

### Cortical–striatal interactions as a key to lateralized processing

Recognition of the plasticity of white matter in response to experience has facilitated new ways of thinking about functional systems in the brain. White matter plasticity has been demonstrated in an increasingly diverse range of motor skills from piano playing (Bengtsson et al., 2005) and juggling (Scholz et al., 2009) to complex balancing (Taubert et al., 2010), occurring over periods spanning a few sessions to decades of practice, usually expressed as increases in FA values. However, the white matter “tuning” for speech in this study likely represents a different process. A left cerebral hemisphere dominant speech system for propositional language generally emerges in right-handed individuals within a critical period during early development without benefit of the training associated with other motor skills (Hensch, 2004). Although older rigid views of a critical period for language were controversial, there are features shared between newer notions of critical periods and the proposed functions of the cortical–striatal connections during speech. These common features include functional competition between inputs, the ability to facilitate or inhibit signals, and structural consolidation of relevant pathways (Hensch, 2004). The asymmetrical tuning of a contralateral cortical–striatal pathway for features of an individual's vocal characteristics echoes the results of the blood flow predictive model for speech rate. The correspondence between functional and structural anatomy suggests that the architecture of brain systems responsible for complex behaviors embodies cortical and subcortical regions connected by white matter tracts specifically tuned for the information being processed. Because of the complexity of language, it can be inferred that these structure–function relationships also play a significant role in switching between languages in multilingualism as well as between modes within a language.

## Conclusions

Complex functions, such as speech, reflect the output of integrated systems of specialized functional and structural anatomy. As with the original functional imaging data on speech rate, the relevant structural characteristics of a cortical–striatal network for motor speech control were not identified based on gross left/right or ipsilateral/contralateral differences, but on relationships between individual differences in both performance and morphological characteristics. Understanding the properties of this simple cortical–striatal network during speech may provide a clearer understanding of the speaking brain as well as new insights into the complex functions of the basal ganglia.
